# Membrane Topological Model of Glycosyltransferases of the GT-C Superfamily

**DOI:** 10.3390/ijms20194842

**Published:** 2019-09-29

**Authors:** Andreia Albuquerque-Wendt, Hermann J. Hütte, Falk F. R. Buettner, Françoise H. Routier, Hans Bakker

**Affiliations:** Institute of Clinical Biochemistry, Hannover Medical School, 30625 Hannover, Germany; Andreia.Albuquerque-Wendt@path.ox.ac.uk (A.A.-W.); Huette.Jan@mh-hannover.de (H.J.H.); Buettner.Falk@mh-hannover.de (F.F.R.B.); Routier.Francoise@mh-hannover.de (F.H.R.)

**Keywords:** glycosyltransferase, *N*-glycosylation, *C*-mannosylation, *O*-mannosylation, dolichol-phosphate, mannose, oligosaccharyltransferase, mannosyltransferase, GPI-anchor, endoplasmic reticulum

## Abstract

Glycosyltransferases that use polyisoprenol-linked donor substrates are categorized in the GT-C superfamily. In eukaryotes, they act in the endoplasmic reticulum (ER) lumen and are involved in *N*-glycosylation, glypiation, *O*-mannosylation, and *C*-mannosylation of proteins. We generated a membrane topology model of *C*-mannosyltransferases (DPY19 family) that concurred perfectly with the 13 transmembrane domains (TMDs) observed in oligosaccharyltransferases (STT3 family) structures. A multiple alignment of family members from diverse organisms highlighted the presence of only a few conserved amino acids between DPY19s and STT3s. Most of these residues were shown to be essential for DPY19 function and are positioned in luminal loops that showed high conservation within the DPY19 family. Multiple alignments of other eukaryotic GT-C families underlined the presence of similar conserved motifs in luminal loops, in all enzymes of the superfamily. Most GT-C enzymes are proposed to have an uneven number of TDMs with 11 (POMT, TMTC, ALG9, ALG12, PIGB, PIGV, and PIGZ) or 13 (DPY19, STT3, and ALG10) membrane-spanning helices. In contrast, PIGM, ALG3, ALG6, and ALG8 have 12 or 14 TMDs and display a C-terminal dilysine ER-retrieval motif oriented towards the cytoplasm. We propose that all members of the GT-C superfamily are evolutionary related enzymes with preserved membrane topology.

## 1. Introduction

**Figure 1:**
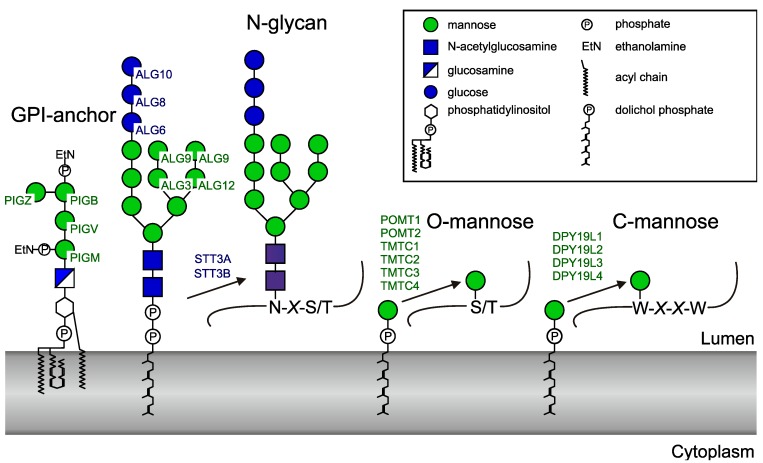
Human enzymes using dolichol-linked donor substrates. The official protein symbols (www.genames.org) of all known human enzymes are indicated. PIG (phosphatidylinositol glycan anchor) enzymes generate the GPI (glycosylphosphatidylinositol)anchor. ALG (asparagine-linked glycosylation) enzymes transfer four mannoses and three glucoses to the lipid-linked oligosaccharide before it is transferred to proteins by the oligosaccharyltransferase complex. STT3 (staurosporine- and temperature-sensitive) is the catalytic subunit of the oligosaccharyltransferase and occurs in two forms. Two POMTs (protein *O*-mannosyltransferases) and four TMTC (transmembrane and tetratricopeptide repeat containing) proteins transfer *O*-mannose to protein. Finally, four homologues of the *Caenorhaditis elegans C*-mannosyltransferase DPY-19 are present in human.

Glycosyltransferases may be classified and named according to the reaction that they catalyze and their substrate specificity. In addition, a classification based on sequence similarities has been adopted and currently organizes glycosyltransferases in 106 distinct CAZy (Carbohydrate Active enZYmes) GT families (http://www.cazy.org/GlycosylTransferases.html) [1]. The majority of these enzymes use nucleotide sugars as donor substrates, whereas others require a polyisoprenoid lipid carrier for presentation of the monosaccharide or oligosaccharide. In eukaryotes, the glycosyltransferases that use dolichol-phosphate (Dol-P)- or dolichol-diphosphate (Dol-PP)-linked substrates act in the endoplasmic reticulum (ER) and are involved in *N*-glycosylation, glypiation, *O*-mannosylation, and *C*-mannosylation of proteins (Figure 1).

Biosynthesis of *N*-glycans involves the assembly of a Dol-PP-linked precursor that typically contains three glucose, nine mannose, and two N-acetylglucosamine residues. Four of the nine mannoses, as well as the three glucoses, are added on the luminal side of the ER membrane by the mannosyltransferases ALG3, ALG9, and ALG12, and glucosyltransferases ALG6, ALG8, and ALG10. Dolichol-phosphomannose (Dol-P-Man) and dolichol-phosphoglucose (Dol-P-Glc) are the donor substrates for these reactions [2]. The oligosaccharide moiety of Glc3Man9GlcNAc2-PP-Dol is then transferred from the lipid anchor to protein by the oligosaccharyltransferase (OST) complex [3]. STT3A or STT3B, two alternative catalytic subunits of the complex, are themselves glycosyltransferases using a dolichol-linked donor but transfer an oligosaccharide instead of a monosaccharide.

Protein glypiation involves the preassembly of a glycosylphosphatidylinositol (GPI) anchor and subsequent transfer to the protein. The four mannosyltransferases, PIGM, PIGV, PIGB, and PIGZ, catalyze GPI-anchor assembly using Dol-P-Man on the luminal side of the ER membrane [4]. Finally, protein-*O*-mannosyltransferases [5,6] and *C*-mannosyltransferases [7,8] use Dol-P-Man to catalyze the transfer of mannose to the hydroxyl group of serine or threonine and to the indole carbon 2 of tryptophan to form *O*-mannose and *C*-mannose, respectively. Like *N*-glycosylation, protein *O*-mannosylation is found in eukaryotes, bacteria, and archea [9], whereas C-mannosylation is restricted to metazoa and alveolata [8,10]. The human genome encodes six *O*-mannosyltransferases, classified into two protein families (POMT1 and POMT2 [11]; and TMTC1, TMTC2, TMTC3, and TMTC4 [5]) and four homologs of the *C*-mannosyltransferase of *C. elegans* DPY-19 (dumpy-19) [8] (DPY19L1 (DPY-19like1), DPY19L2, DPY19L3, and DPY19L4). Recently, DPY19L1 and DPY19L3 were shown to be active enzymes with different specificities, whereas no activity could yet be demonstrated for DPY19L2 and DPY19L4 [12].

All the above-mentioned enzymes are multitransmembrane proteins with, based on previous data, 7 to 13 predicted or experimentally defined transmembrane domains (TMDs). They have been categorized as enzymes of the GT-C superfamily, primarily because of their multitransmembrane nature and to distinguish them from enzymes with well-defined GT-A and GT-B structural folds [13,14]. Besides the common polyisoprenol phosphate-bound substrates, they possess a catalytic mechanism leading to an inversion of the transferred monosaccharide anomery (inverting glycosyltransferases). However, they exhibit low or nondetectable sequence identity and have thus been assigned to distinct CAZy GT families (Table 1).

Experimental information about the structure and membrane topology of integral membrane glycosyltransferases is still sparse [15]. Previous studies underlined similarity in the overall predicted topology of ALG (asparagine-linked glycosylation) and PIG (phosphatidylinositol glycan anchor) proteins [14,16] and described the presence of a D×D or DD motif, in which aspartic acid (D) can also be replaced by a glutamic acid (E). This motif is located shortly after the first TMD in the predicted first luminal loop [14,17,18] and has been shown to be essential for enzymatic activity in various members of the GT-C superfamily [19,20,21,22,23,24].

Using protein tagging, the catalytic subunit of yeast Stt3 and protein O-mannosyltransferase Pmt1 were suggested to display 11 and seven TMDs, respectively, whereas prediction programs suggested the presence of 13 and 11 TMDs [25,26,27,28]. The structures of bacterial and archaeal OSTs named PglB (protein glycosylation B) and AglB (archaeal glycosylation B) and of the yeast STT3 demonstrated that these enzymes have 13 membrane helices [21,24,29], and a recent crystallization study of the *O*-mannosyltransferase revealed that this enzyme has 11 TMDs [30]. However, the defined topology of PglB/AglB proteins differs from the 13 TMD models obtained by algorithms, which predicted a TMD in the first external loop (EL1) (localized in the periplasm in bacteria) and failed to detect the fifth transmembrane helix. In contrast, experimental topology mapping of the yeast STT3 correctly predicted the first luminal loop despite its hydrophobicity, but missed two other domains [26].

Previously, we described clear similarities between the OST catalytic subunit and *C. elegans C*-mannosyltransferase DPY-19 that suggests an evolutionary link between these enzymes [8]. In this work, we generated a membrane topology model of DPY19 family proteins based on TMDs prediction and information provided by the crystal structure of PglB and AglB, and analyzed the conservation of amino acids in the predicted loops. We strengthen this topology model by demonstrating the importance of several conserved luminally oriented amino acid residues for *C*-mannosylation of proteins. We further propose a consistent membrane topology model for all eukaryotic members of the GT-C superfamily.

## 2. Results and Discussion

### 2.1. Membrane Topology Model of DPY19 Proteins

**Figure 2:**
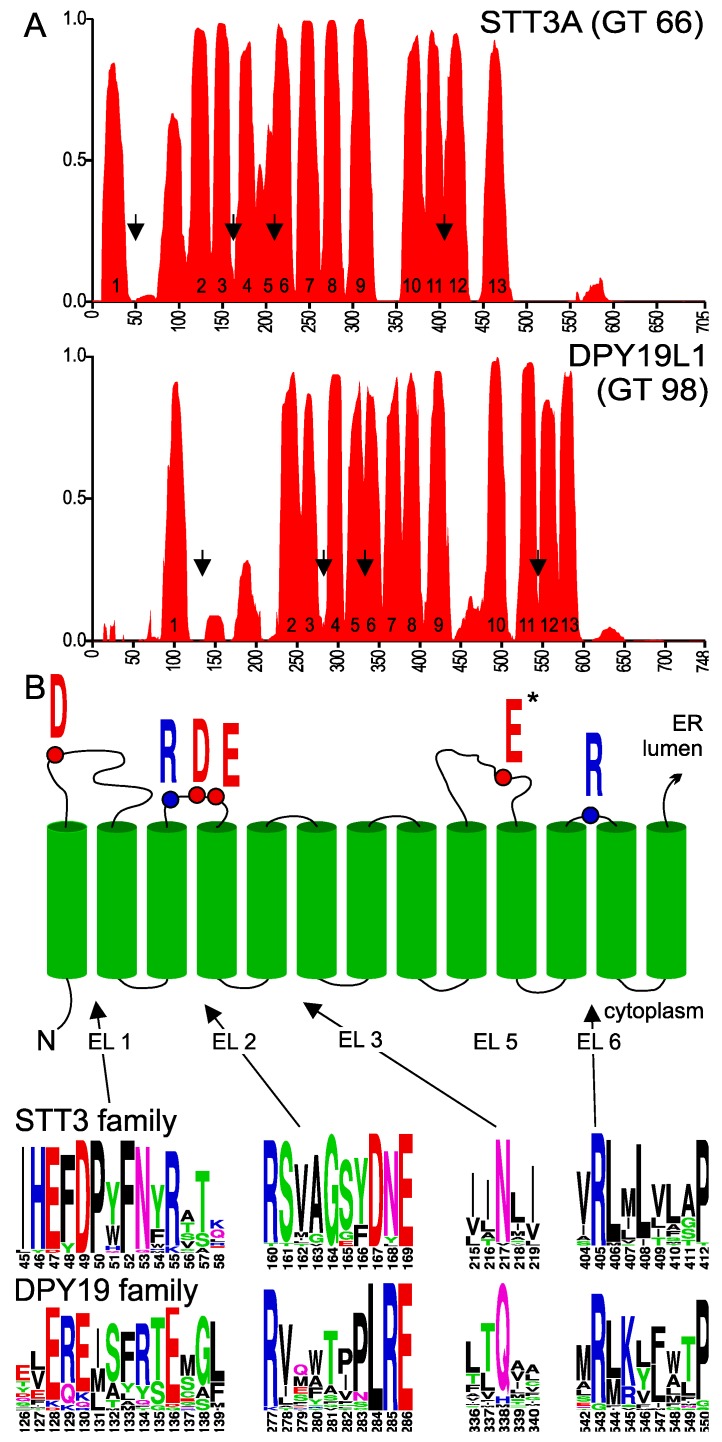
(**A**) Transmembrane probability of STT3A and DPY19L1. Transmembrane domain prediction was carried out using the algorithm PolyPhobius with a multiple alignment comprising 25 most diverse members of the two families, as described in experimental procedures. Proposed transmembrane domain (TMD) numbering is based on the structure of bacterial *Campylobacter lari* oligosaccharyltransferase [21]. (**B**) Upper panel: Conserved residues involved in catalysis in STT3. Lower panel: Conserved areas (arrows in Figure 2A) in extracytoplasmic (luminal) loops (EL) of the STT3 and DPY19 family. *E in EL5 is part of the catalytic pocket, but due to the large size of this loop and the presence of multiple D (aspartic acid) and E (glutamic acid) residues, no clear equivalent can be observed in DPY19 enzymes (see supplemental Figure S1A,B). The asparagine (N) in EL3 is highly conserved in STT3, but has not been associated with the catalytic pocket.

Membrane topology prediction of members of the GT-C family is rather inconsistent. Enzymes of different species often show distinct patterns of TMD probability. To partly overcome this variation, we used the program PolyPhobius (http://phobius.sbc.su.se/) [25]. This algorithm can be supplied with a multiple sequence alignment and predicts TMDs of the first sequence in the alignment (in our case, the human enzyme STT3A or DPY19L1) but bases the prediction on the multiple sequence alignment. At least 25 eukaryotic STT3 and DPY19 homologs were used to generate multiple sequence alignments. These sequences were selected so that the evolutionary most distinct eukaryotic species are represented. TMD probability of human STT3A and DPY19L1 was compared (Figure 2). The topology model of STT3A has been annotated using information provided by the crystal structures of PglB/AglB and yeast STT3 [21,24,29]. Residues 38 to 114 of STT3A were thus interpreted as the first luminal loop despite the high hydrophobicity of part of this region. Likewise, the large hydrophobic region encompassing residues 200 to 230, predicted as one domain by the program, was marked as TMDs 5 and 6. Annotation of the membrane-spanning domains of DPY19L1 was based on comparison with STT3A and corresponds to the 13 domains previously predicted for the *C. elegans* enzyme [8]. In spite of low sequence identity between STT3 and DPY19 enzymes, the transmembrane prediction profile of DPY19L1 concurred perfectly with the STT3A model (Figure 2). In DPY19 proteins, the hydrophobicity in the first loop is lower than in STT3 and the 5th and 6th membrane domain are distinguishable, showing a perfect agreement between in silico prediction and the annotation based on the homology to OSTs.

In order to identify amino acids residues potentially involved in *C*-mannosyltransferase activity, we analyzed the conservation of amino acids in DPY19 proteins using the multiple sequence alignment generated for transmembrane prediction. We produced sequence logos (weblogo.berkeley.edu) [31] of the multiple alignments (Figure S1A,B), in which we maintained the numbering of human STT3A and DPY19L1. Comparison of the logo graphs and the annotated TMDs revealed conservation of amino acids in luminal domains (after uneven TMDs), but not in cytoplasmic domains (Figure S1A,B). In *Campylobacter lari* PglB, four external (or extracytoplasmic) loops (EL) contain catalytic residues D56 (EL1), R147, D154 and D156 (in EL2), E319 (in EL5), and R375 (in EL6) [21]. These active site amino acids are conserved in archaea AglB, with the exception of D156 that is replaced by a histidine [29], and in eukaryotic STT3s. In human STT3A, the amino acids D49, R160, D167, E169, E351, and R405 have been proposed to be catalytic residues [21,24,29].

DPY19 proteins contain an ExE motif that is located shortly after the first TMD and precedes the hydrophobic stretch of EL1, like the ExD motif of STT3s (Figure 2B). A conserved Rx_6_DNE or Rx_6_LRE motif was identified in EL2 of eukaryotic STT3s and DPY19s, respectively. As shown below, the Rx_6_LRE motif is essential for DPY19 function but was proposed to be located on the cytoplasmic site in a recent topology model of DPY19L3 [32]. This is rather unlikely and probably reflects the limits of the experimental approach using luciferase fusion proteins. The 3rd TMD is entirely embedded in the protein in STT3 and might not function as a transmembrane domain without the presence of the following domains.

The large EL5 of PglB/AglB contains a glutamate (E) interacting with both a divalent cation and the asparagine acceptor of these enzymes. In DPY19 proteins, four to six acidic residues are conserved in EL5, but none seems completely conserved (Figure S1B). Finally, as in STT3, the short EL6 of DPY19s exhibits a highly conserved arginine (R) residue followed by a Proline (P) embedded in transmembrane domain 12 (Figure 2B). In the large C-terminal luminal domain, no conservation between STT3 and DPY19 enzymes was observed. This is unsurprising, since this part of the protein has been shown to be involved in protein acceptor substrate binding in STT3 [33].

### 2.2. Importance of Conserved Amino Acids for C-Mannosylation

**Figure 3:**
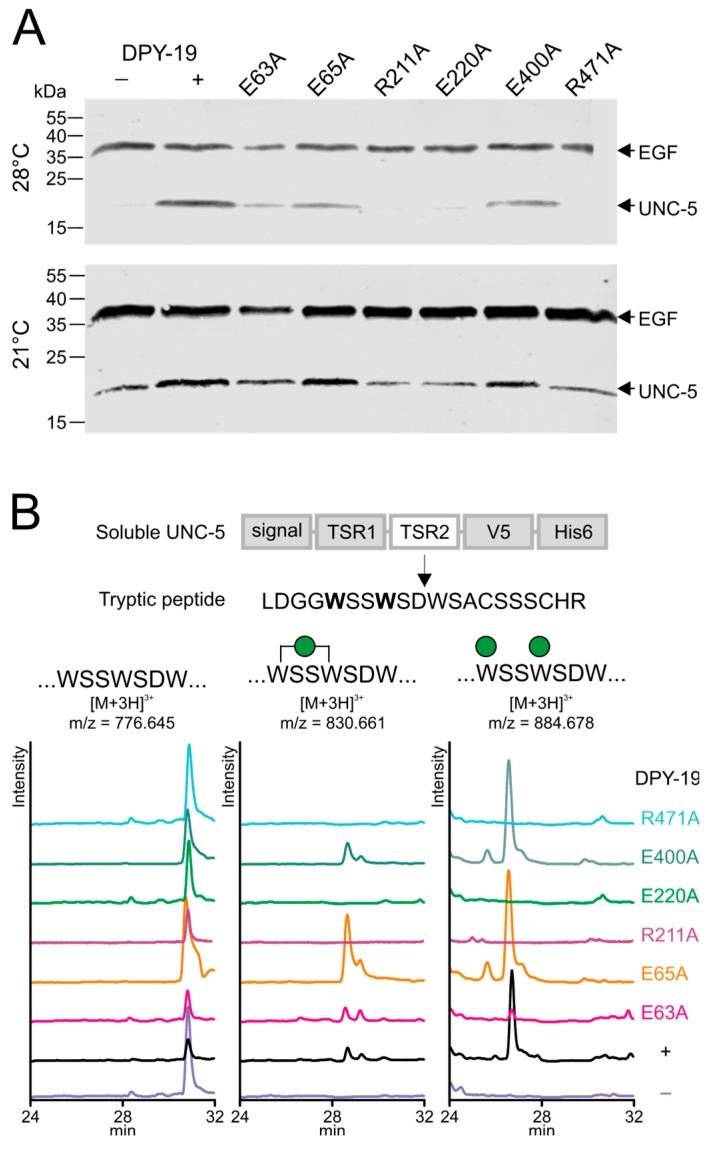
Secretion and *C*-mannosylation of an UNC-5 fragment modified by native and mutated *C. elegans* DPY-19. (**A**) Secretion of UNC-5 from S2 cells grown at 21 and 28 °C. A V5-His_6_-tagged UNC-5 construct encompassing the two TSRs was transfected in S2 cells without (−) or with (+) *C. elegans* DPY-19 or with *C. elegans* DPY-19 carrying the mutation E63A, E65A, R211A, E220A, E400A, or R471A. A plasmid expressing V5-tagged epidermial growth factor (EGF)-repeats 16–20 (EGF) of *Drosophila melanogaster* Notch was used as a transfection and secretion control. Transfected cells were grown at 21 or 28 °C, and secreted proteins were detected on Western blot using an anti-V5 antibody. (**B**) Extracted ion chromatograms of the unmodified, mono-*C*-mannosylated, and di-*C*-mannosylated TSR2 tryptic peptide LDGGWSSWSDWSACSSSCHR. All graphs are shown with equal intensities (13,000 counts per second).

We have previously reported the importance of *C*-mannosylation for stability and transport of the mouse netrin receptor UNC5 to the cell surface [12]. When a soluble *C. elegans* UNC-5 construct containing the two thrombospondin repeats was expressed in *Drosophila* Schneider 2 (S2) cells, the non-*C*-mannosylated protein fragment was secreted and could be purified from the culture medium when the cells were grown at 21 °C (Figure 3A, lower panel, lane 1). In contrast, when the insect cells were grown at 28 °C, UNC-5 could be obtained from the culture medium only if *C. elegans C*-mannosyltransferase DPY-19 was co-transfected (Figure 3A, upper panel lanes 1 and 2). This system was used to assess the importance of the above-mentioned conserved residues for *C. elegans* DPY-19 activity. The amino acids residues E63, E65, R211, E220, E400, and R471 of DPY-19 were individually mutated to alanine, and the resulting enzymes were expressed in S2 cells with UNC-5. Protein secretion at 21 and 28 °C was analyzed by Western blot (Figure 3A). At 28 °C, UNC-5 was secreted when expressed in the presence of DPY-19 carrying the mutations E63A, E65A, or E400A, suggesting that these mutations only had a partial or no effect on C-mannosylation. In contrast, the mutations R211A, E220A, and R471A inactivated DPY-19 since no UNC-5 was secreted when transfected cells were grown at 28 °C. To substantiate these results, UNC-5 purified from medium of cells cultivated at 21 °C was digested with trypsin, and the *C*-mannosylation degree of the resulting peptides was analyzed by liquid chromatography–mass spectrometry (LC-MS). As previously reported [8], an abundant [M+3H]^3+^ ion at m/z 884.68, corresponding to the tryptic peptide LDGGWSSWSDWSACSSSCHR substituted with two mannoses was observed when UNC-5 was modified by wild-type DPY-19 (Figure 3B). Triple-charged ions at m/z 776.65 and 830.66 of the same peptide with zero and one mannose, respectively, were of much lower intensity. A very similar profile was observed when UNC-5 was purified from cells expressing the mutant protein E65A and E400A, confirming that these mutations had no significant effect on DPY-19 activity. UNC-5 modified by the mutant proteins E63A yielded reduced di-mannosylated peptide, indicating reduced *C*-mannosylation activity. The conserved E63 and E65 might be redundant, and mutations at most reduce *C*-mannosylation activity, as seen for several other GT-C enzymes [19,22]. Cells transfected with an empty vector or with a construct encoding the mutant protein R211A, E220A, or R471A showed no sign of *C*-mannosylation (Figure 3B). These results indicate that the latter residues, conserved between STT3 and DPY19 proteins, are essential for *C*-mannosylation. Most likely, these amino acids have the same function in STT3 and DPY19 enzymes in the reaction mechanism, but we cannot exclude an influence on expression or localization of the enzyme.

### 2.3. Membrane Topology Model of Other GT-C Family Members

Since all eukaryotic glycosyltransferases belonging to the GT-C superfamily use Dol-P- or Dol-PP-linked donor substrates, proceed via an inverting mechanism, and require a divalent cation, we assumed similarities between these proteins and considered the possibility to extend the STT3/DPY19 topology model to other GT-C proteins. Previous studies already underlined similarity in the overall topology of the ALG and PIG proteins [16] and created a membrane topology model with 12 TMDs.

Some sequences of ALG and PIG enzymes can be aligned. ALG9, ALG12, PIGB, and PIGZ are grouped together in the GT22 family. ALG3 and PIGM show clear homology with each other as well, although they are placed in different families (GT58 and GT50, respectively). The glucosyltransferases ALG6 and ALG8 (GT57) are very similar but differ from the 3rd glucosyltransferase (ALG 10, GT59). PIGV (GT76) also forms a separate family. Besides the presence of a D×D or similar motif [14,16], the whole ALG and PIG families of enzymes cannot directly be aligned. The same is true for the *O*-mannosyltransferases, POMT1 and -2 (GT39) and the recently identified TMTC (GT105) family [5].

**Figure 4:**
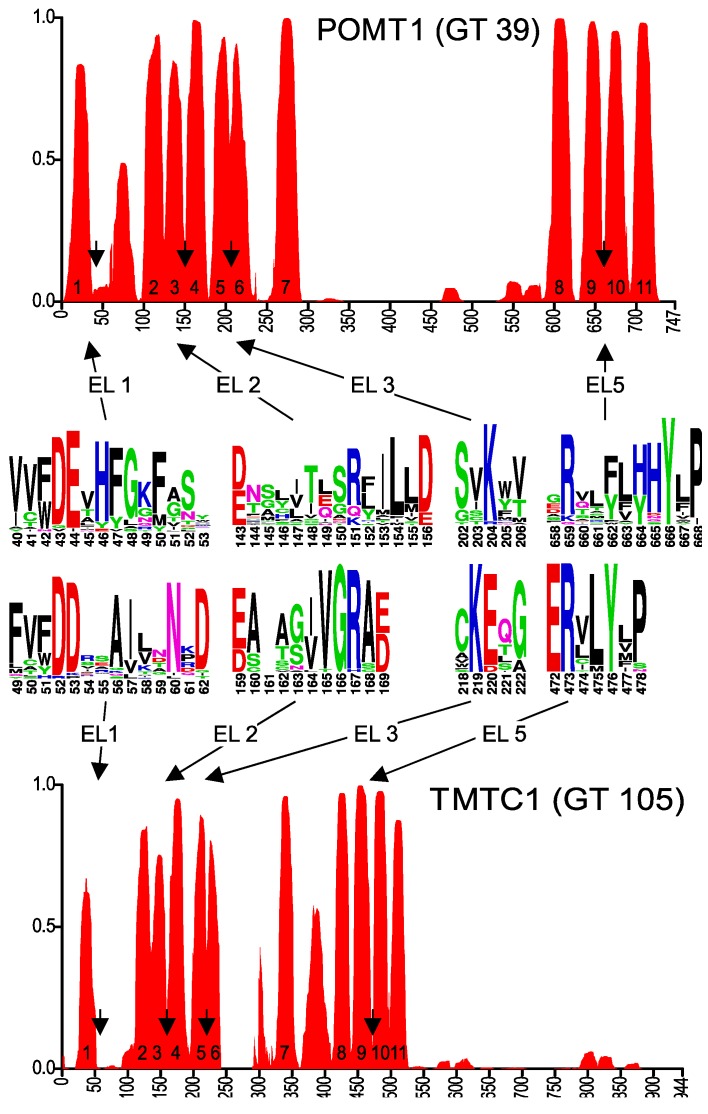
Predicted membrane topology and amino acid conservation of human POMT1 and TMTC1. Transmembrane domain prediction was carried out using the algorithm PolyPhobius, as described in experimental procedures. The position of the predicted extracytoplasmic loops EL1, EL2, EL3 and EL5 is indicated by arrows and amino acid conservation in these loops is shown in the sequence logos.

To identify conserved motifs and putative membrane topology, we selected the 25 to 30 most diverse eukaryotic sequences of each GT-C glycosyltransferase family and used these to generate transmembrane probability graphs and sequence logos, as described above for DPY19. The transmembrane prediction graph of the POMT family (Figure 4) clearly showed common characteristics with those of STT3 and DPY19 (Figure 2). As in these two enzymes, there is a larger luminal loop after the first TMD, followed by an area with TMDs with small loops. Before the last four TMDs, there is again a larger loop. A conserved Rx_4_D, similar to the motif found in STT3 and DPY19 proteins, was found between predicted TMDs 3 and 4. As in STT3, domains 5 and 6 were considered as two TDMs separated by a short luminal loop, which displays a highly conserved lysine (K). In POMTs, the conserved Rx_6_P motif situated before the last two transmembrane domains, described above for STT3s and DPY19s, is present as well, however, with eight amino acid distance (Figure 4).

The topology prediction of POMT can perfectly be aligned with STT3 and DPY19, with the conserved motifs at identical positions, although two TMDs less are predicted in the middle part (Figure 4). Based on the conservation in the first luminal loop, conserved catalytic mechanisms between *O*-mannosyltransferases and other members of the GT-C superfamily were already suggested by Lommel et al. [22]. Using experiments with hybrid proteins, the yeast *O*-mannosyltransferase Pmt1 was originally proposed to have seven TMDs [28]. The crystal structure of yeast *O*-mannosyltransferase Pmt1 and Pmt2 that was just reported [30] is, however, in complete agreement with our proposed 11 TMD model for POMT. It also highlights the role of the conserved lysine (K) in EL3 (K204 in POMT1, Figure 4) and R649 in EL5 in Dol-P-Man binding and the structural resemblance with STT3.

TMTC proteins also have a similar TMD prediction (Figure 4), resembling POMT in the number of predicted domains, but with a long C-terminal luminal domain like in STT3 and DPY19. A multiple alignment of diverse members of the TMTC family again reveals conserved areas resembling those of POMT (Figure 4 and Figure S1D).

### 2.4. GT-C Glycosyltransferases Involved in N-Glycan and GPI Precursor Biosynthesis

**Figure 5:**
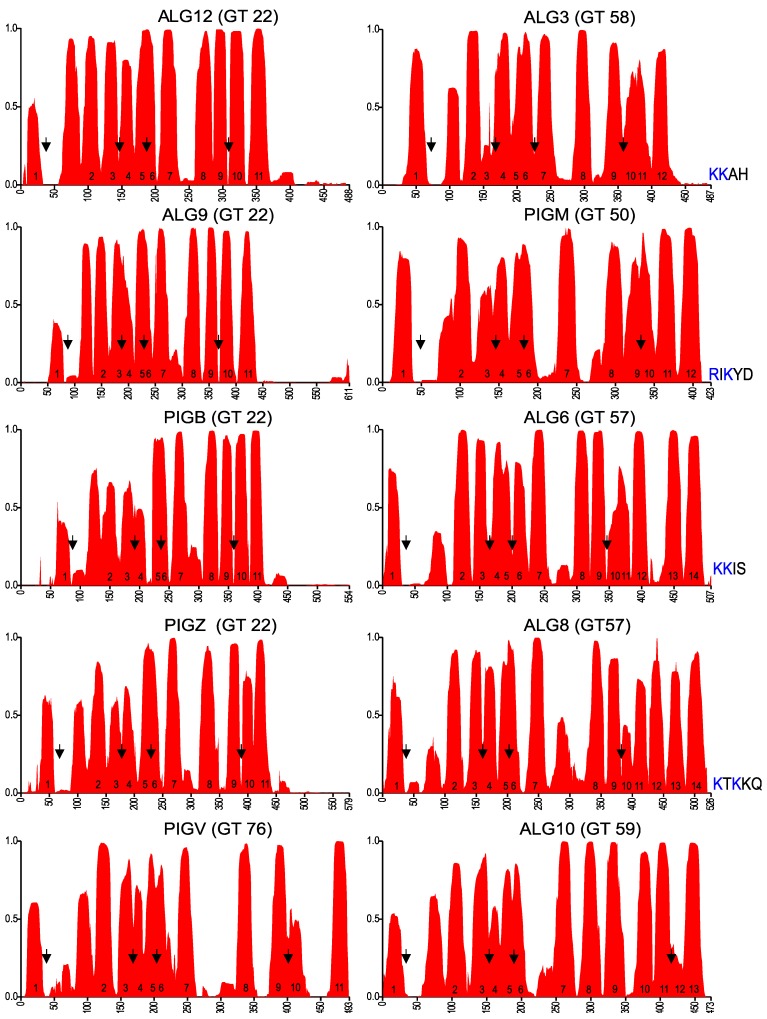
Membrane topology prediction of other eukaryotic glycosyltransferases of the GT-C superfamily. Arrows indicate the positions of the conserved areas that are depicted in Figure 6, corresponding to EL1, EL2, EL3, and EL5 (EL6 for ALG10, due to the two additional TMDs). The dilysine motif at the C-terminus of proteins with even numbers of predicted TMDs is shown.

**Figure 6:**
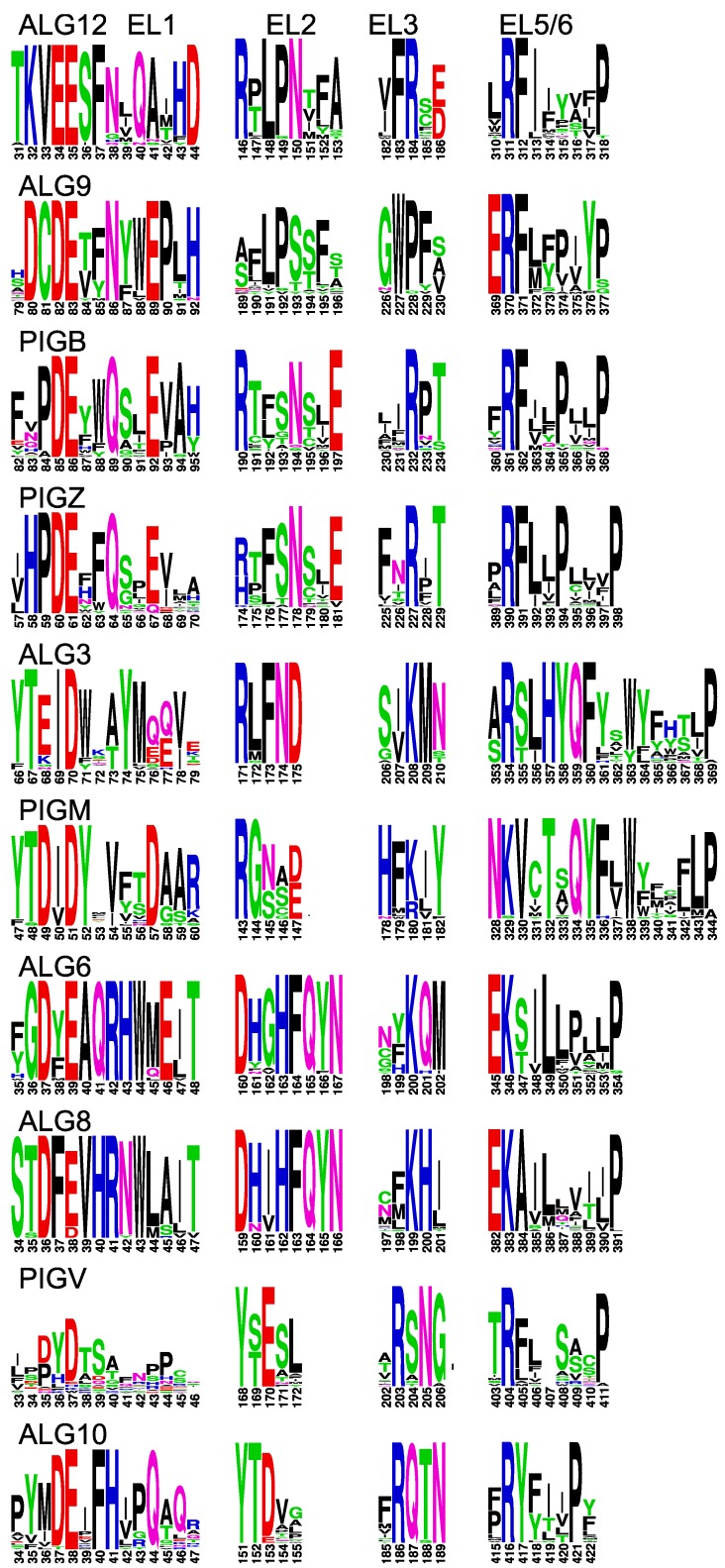
Conserved domains in extracytoplasmic loops of GT-C enzymes. Conserved domains in EL1, EL2, EL3, and EL5 (EL6 for ALG10) of indicated GT-C members, presented as logos. The positions correspond to arrows in Figure 5.

The enzymes analyzed so far use proteins as the acceptor substrate. The remaining members of the GT-C superfamily transfer lipid-linked sugars to a lipid-linked acceptor. These enzymes, however, show again a comparable TMD prediction (Figure 5). With few exceptions, four conserved amino acid patterns can be found in the multiple alignments of all enzymes (Figure 6 and Figure S1E–N). The GT22 enzymes and PIGV are predicted to have 11 TMDs (Figure 5), corresponding to those in the *O*-mannosyltransferases (Figure 4). The conserved patterns in ALG10 align with STT3 and DPY19 (Figure 2), suggesting an identical 13 TMD model. ALG3 and PIGM show 12 TMDs. The 12th domain is, based on the positions of the conserved domains, a C-terminal addition compared to the enzymes with 11 TMDs and displays a dilysine motif [16]. The 12 TMDs model places this motif on the cytoplasmic side of the membrane where it can function as an ER-retrieval signal [34]. ALG6 and ALG8 display three additional TMDs at the C-terminus, thus comprising 14 TMDs, and contain a dilysine motif as well.

The concept that conserved residues should be located on the luminal side of the membrane, where the catalyzed reaction takes place, helped to create topology models of all enzymes of the GT-C superfamily. Four conserved amino acid patterns can be observed in luminal loop 1, 2, 3, and 6(or 5) in all enzymes (Figure 2,Figure 4 and Figure 6). These are not identical among the different GT-C enzymes, but show similar characteristics. The first D×D motif, in which D can be E and x may be absent, is present in all enzymes. The second conserved pattern is more variable, but mostly contains an R and/or D/E/N or Q. The motif of the 3rd luminal loop comprises mostly one conserved positively charged R or K, but is replaced by N or Q in STT3 and DPY19 (Figure 2). ALG9 is an outlier, in which the motifs of the 2nd and 3rd luminal loop are absent. Possibly, these potential catalytic residues are compensated for in other luminal loops.

The most striking conservation is the presence of an R or K in the luminal loop of the cluster of four TMDs, which are at the C-terminus in most enzymes. This conserved positively charged amino acid, predicted to be involved in donor substrate binding [21,30], is always followed by a conserved P that form an Rx_4–14_P motif. In addition, the large area between TMD 9/10 (or 7/8) has conserved amino acids in all families (Figure S1) that are potentially involved in catalysis, like in STT3. This area is too large and variable to identify patterns common to the whole superfamily, but the presence of conserved amino acids within families is a strong argument for luminal localization. Finally, in most enzymes the C-terminus is oriented toward the ER lumen and also contains conserved amino acids within the families.

The types of conserved amino acids in the GT-C family are those anticipated to be involved in catalysis. To ascertain that these are not the types of pattern always found on luminal sides of membrane proteins, we carried out the same analysis with an ER transmembrane protein that catalyzes a reaction on the cytoplasmic side. The 10 TMD protein dolichyl-phosphate N-acetylglucosaminephosphotransferase (DPAGT1), which has been crystalized [35], indeed shows major conservation in loops on the cytoplasmic side of the membrane (Figure S1O).

## 3. Materials and Methods

### 3.1. Bioinformatics

Using the human protein sequences of members of the GT-C family (Figure 1), a PSI-BLAST (Position-Specific Iterative Basic Local Alignment Search Tool) [36] search was performed in the reference protein database, restricted to eukaryotic species. At least 25 most diverse sequences were selected for each protein. For each protein, paralogs (STT3B; DPY19L2, 3, and 4; POMT2; and TMTC2, 3, 4, and various fungal *O*-mannosyltransferasess) were included. These sequences were used for multiple alignments using the program Praline (ibi.vu.nl/programs/pralinewww) that considers transmembrane prediction in its alignment [37]. These multiple alignments were subsequently used to predict the membrane topology using PolyPhobius (phobius.sbc.su.se/poly.html), which allows to plot membrane topology probability of a protein, but based on a multiple alignment [25], allowing a more reliable prediction.

The same multiple alignments were used to generate a protein sequence logo (weblogo.berkeley.edu/) [31]. For the logos, sequences of the human enzymes (in case of paralogs, the lowest number) were taken as basis, meaning that inserted amino acids present in some other sequences were deleted from the alignment before the logo was generated, to avoid gaps and to maintain the numbering of the human enzyme.

### 3.2. Plasmid Constructs for S2 Cell Expression

All *C. elegans* DPY-19 mutants were created using a previously described plasmid: pIB-DPY-19 [8]. DPY-19-E63A, DPY-19-E65A, DPY-19-R211A, DPY-19-E220A, DPY-19-E400A, and DPY-19-R471A were made by amplifying two PCR fragments extended by recognition sequences for the type IIs restriction enzyme BbsI [38] containing the mutated site, which were, after digestion, ligated back in the same vector. The Netrin receptor UNC-5 construct pMT-UNC-5 is described in [8]. As an internal transfection and secretion control, a plasmid expressing EGF repeats 16–20 of *Drosophila melanogaster* Notch (*pMT*-*EGF*), which is described in [39], was used.

### 3.3. Protein Expression in S2 Cells

*Drosophila* Schneider (S2) cells were grown in Insect-XPRESS Protein-free Medium (Lonza Basel, Switzerland) and kept at a density between 3 × 10^6^ and 2 × 10^7^ cells/mL at 24 °C under 30 rpm rotation in 75 cm^2^ nonadherent T-flasks. Transient expression was carried out as described [8] in S2 cells in 24-well plates. An amount of 0.5 µg of *pIB-DPY-19* (wild-type or mutants) or empty vector was combined with 0.5 µg of *pMT-UNC-5* and 0.5 µg of *pMT-EGF*. After Six hours, expression was induced with 0.2 mM CuSO_4_, and cells were subsequently kept at 21 °C under rotation or at 28 °C for 4 days. To produce protein for mass spectrometry (MS) analysis, the protocol was scaled up to 10 mL in 75 cm^2^ T-flasks.

### 3.4. Western Blotting

For western blot analysis, 50 µL of cell suspension was centrifuged at 300× g for 5 min to pellet the cells, followed by a second centrifugation of the supernatant at 4000× g. Ten µL of 5× Laemmli with 5% (*v*/*v*) β-mercaptoethanol was added to 40 µL of the supernatant and incubated at 95 °C for 10 min. The entire sample was loaded on a 15% (*w*/*v*) SDS-PAGE gel. Blotted nitrocellulose membranes were incubated with mouse V5 antibody (Acris Antibodies GmbH, Herford, Germany) and IRDye 800 goat antimouse secondary antibody (LI-COR BioSciences GmbH, Bad Homburg, Germany) and scanned on an LI-COR Odyssey Infrared Scanner.

### 3.5. Protein Purification

For nickel affinity chromatography of UNC-5, 10 mL of medium were centrifuged as described above and filtered through a 0.2 µm membrane (Waters GmbH, Eschborn, Germany). Binding buffer was added to generate 50 mL of 500 mM NaCl, 20 mM Tris-Hcl (pH8), and 20 mM imidazole (buffer A). The solution was loaded on a 1 mL HisTrap HP column (GE Healthcare Europe GmbH, Freiburg, Germany), washed with 10 mL of buffer, and eluted with a 7 mL gradient from 20 to 500 mM imidazole in buffer A. The peak fractions (A280) were precipitated with 4 volumes of acetone, pooled, and resuspended in 30 µL of 1× Laemmli (without reducing agents). Finally, 1 µL of the preparation was used for western blotting, and the rest of the sample was loaded on a 15% (*w*/*v*) SDS-PAGE and stained with Coomassie blue for MS analysis.

### 3.6. Mass Spectrometry

Protein bands were excised from gels and digested with trypsin at 37 °C in an overnight reaction. Peptides were extracted and analyzed by mass spectrometry, as described [8].

## 4. Conclusions

**Figure 7:**
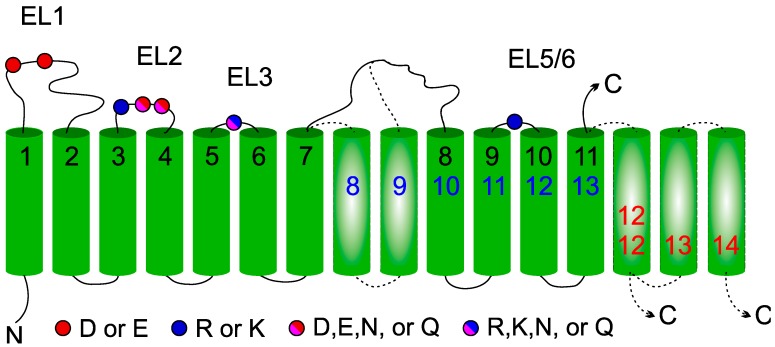
General membrane topology model of glycosyltransferase-C members. Location of the four conserved extracytoplasmic loops in the GT-C families are indicated by color-coded balls representing acidic amino acids. The 11 TMDs (numbered in black) present in all GT-C members are depicted. Inserted TMDs (in broken lines) increase the number of TMDs to 13 (blue) or to 12/14 with the C-terminus in the cytoplasm (red).

In conclusion, we can state that all enzymes of the GT-C superfamily are part of a true homologous family, as was suggested before [14,16,22]. Amino acids involved in STT3 catalysis can mostly be found in other GT-C enzymes. The conservation of charged R/K and acidic D/E amino acid residues suggests common donor substrate recognition and catalysis involving amino acids from different luminal loops. All members of the GT-C family have a similar topological organization (Figure 7). The majority of enzymes present 11 TMDs (POMTs, TMTCs, ALG12, ALG9, PIGB, PIGZ, and PIGV) with the N-terminus oriented toward the cytoplasmic face of the ER membrane and the C-terminus facing the lumen. The first and fourth luminal loops are relatively large and connect the first and four last TMDs to six central TMDs (1-6-4 organization). In the 13 TMDs model adopted by STT3s, DPY19s, and ALG10, two additional central membrane segments are observed, resulting in a 1-8-4 organization. Finally, one (ALG3, PIGM) or three (ALG6 and ALG8) extra C-terminal TMDs give rise to 12 or 14 TMDs (1-6-5 or 1-6-7 organization). In these enzymes, the dilysine-containing C-terminus is oriented toward the cytoplasm, where it can act as retrieval signal.

## Figures and Tables

**Table 1:** Assignment of human glycosyltransferases that uses dolichol linked substrate to CAZy glycosyltransferase families.

Enzymes	GT Family
ALG9, ALG12, PIGB, PIGZ	GT22
POMT1, POMT2	GT39
PIGM	GT50
ALG6, ALG8	GT57
ALG3	GT58
ALG10	GT59
STT3A, STT3B	GT66
PIGV	GT76
DPY19L1, DPY19L2, DPY19L3, DPY19L4 TMTC1, TMTC2, TMTC3, TMTC4	GT98 GT105

## Supplementary Materials

The following are available online at https://www.mdpi.com/1422-0067/20/19/4842/s1, Figure S1: Full multiple alignments presented as logos of all GT-C families.

## Abbreviations

CAZy	carbohydrate active enzymes
Dol	dolichol
ER	endoplasmic reticulum
EL	extracytoplasmic loop
Glc	glucose
GlcNAc	N-acetylglucosamine
GT-C	glycosyltransferase family C
GPI	glycosylphosphatidylinositol
Man	mannose
OST	oligosaccharyltransferase
TMB	transmembrane domain
