# Supplementary material for: Membrane Topological Model of Glycosyltransferases of the GT-C Superfamily
Source: Int J Mol Sci. 2019 Sep 29;20(19):4842. doi: 10.3390/ijms20194842 (PMC6801728; doi:10.3390/ijms20194842)
Supplement: Supplementary file 1 [file ijms-20-04842-s001.pdf]

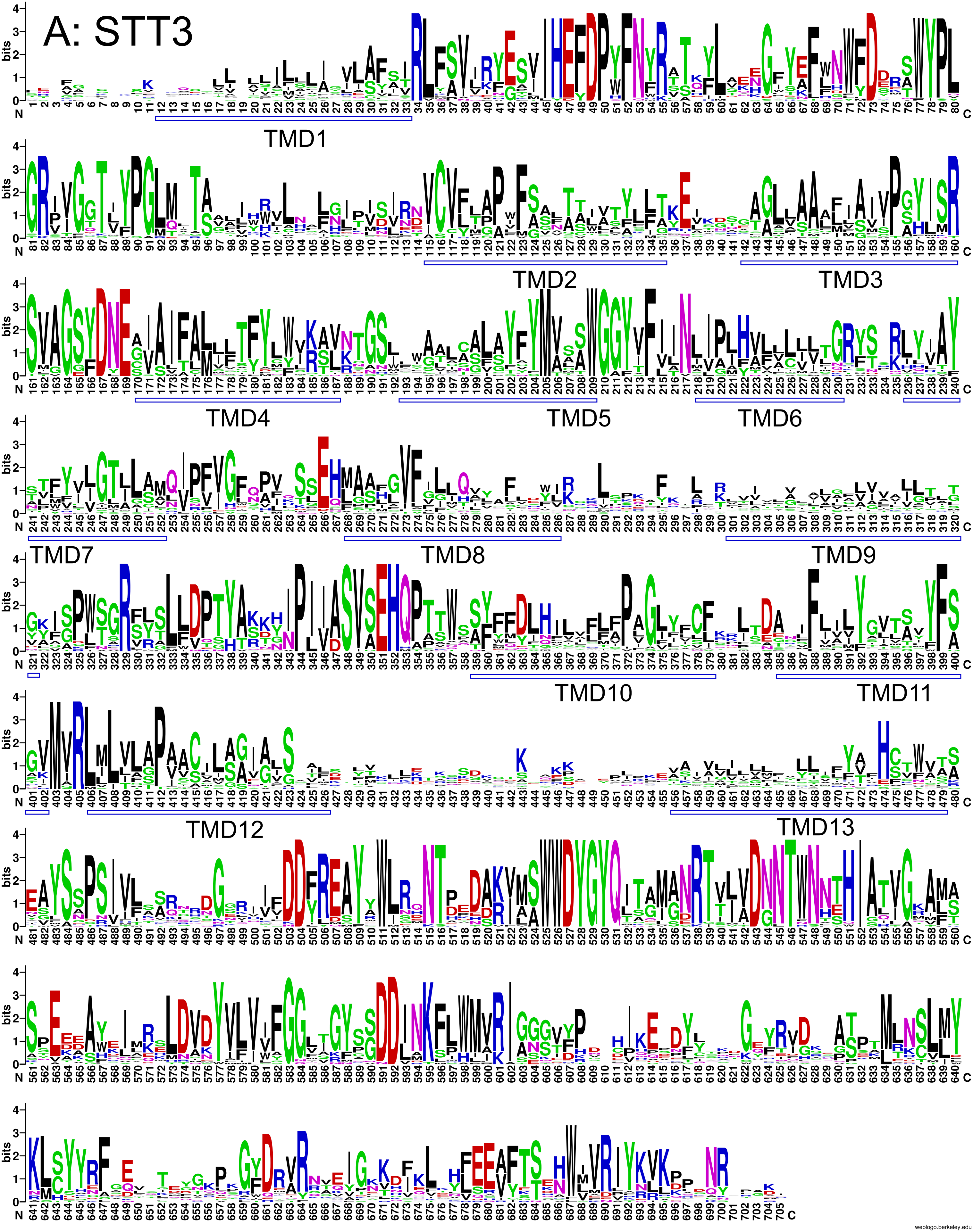
A: STT3
bits
TMD1
TMD2
TMD3
TMD4
TMD5
TMD6
TMD7
TMD8
TMD9
TMD10
TMD11
TMD12
TMD13
weblogo.berkeley.edu

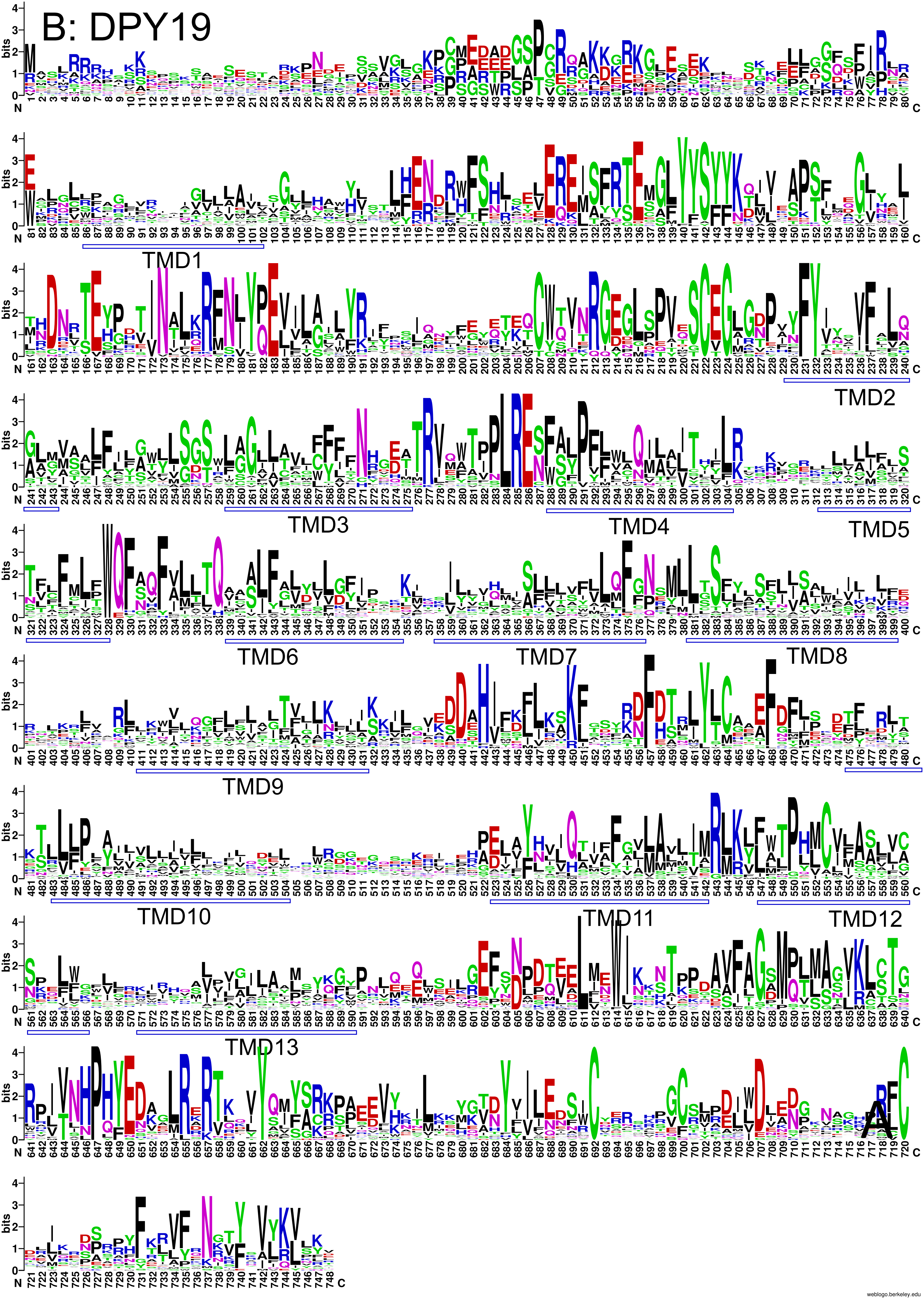
B: DPY19
TMD1
TMD2
TMD3
TMD4
TMD5
TMD6
TMD7
TMD8
TMD9
TMD10
TMD11
TMD12
TMD13
weblogo.berkeley.edu

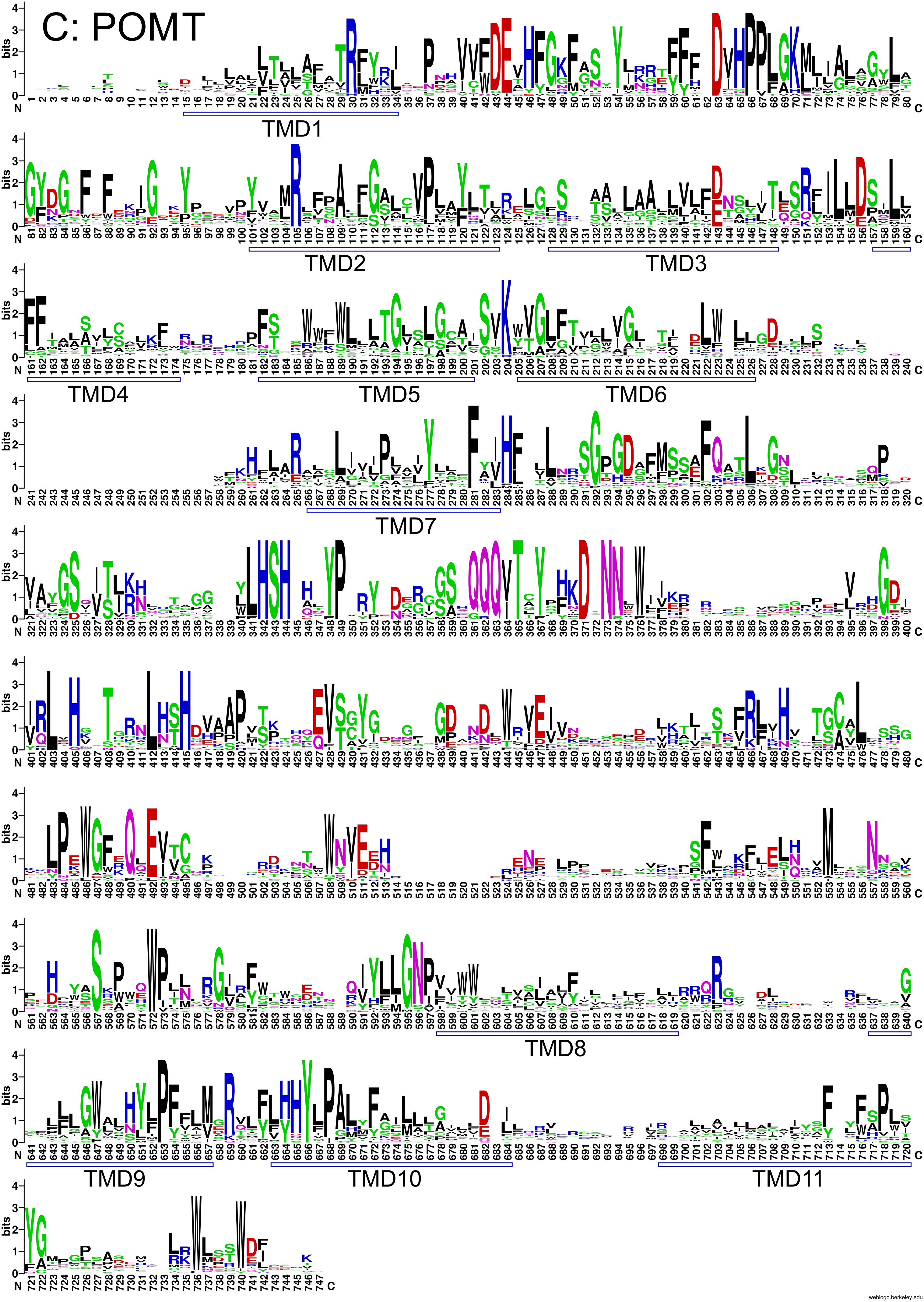
C: POMT
bits
N
C
TMD1
TMD2
TMD3
TMD4
TMD5
TMD6
TMD7
TMD8
TMD9
TMD10
TMD11
weblogo.berkeley.edu

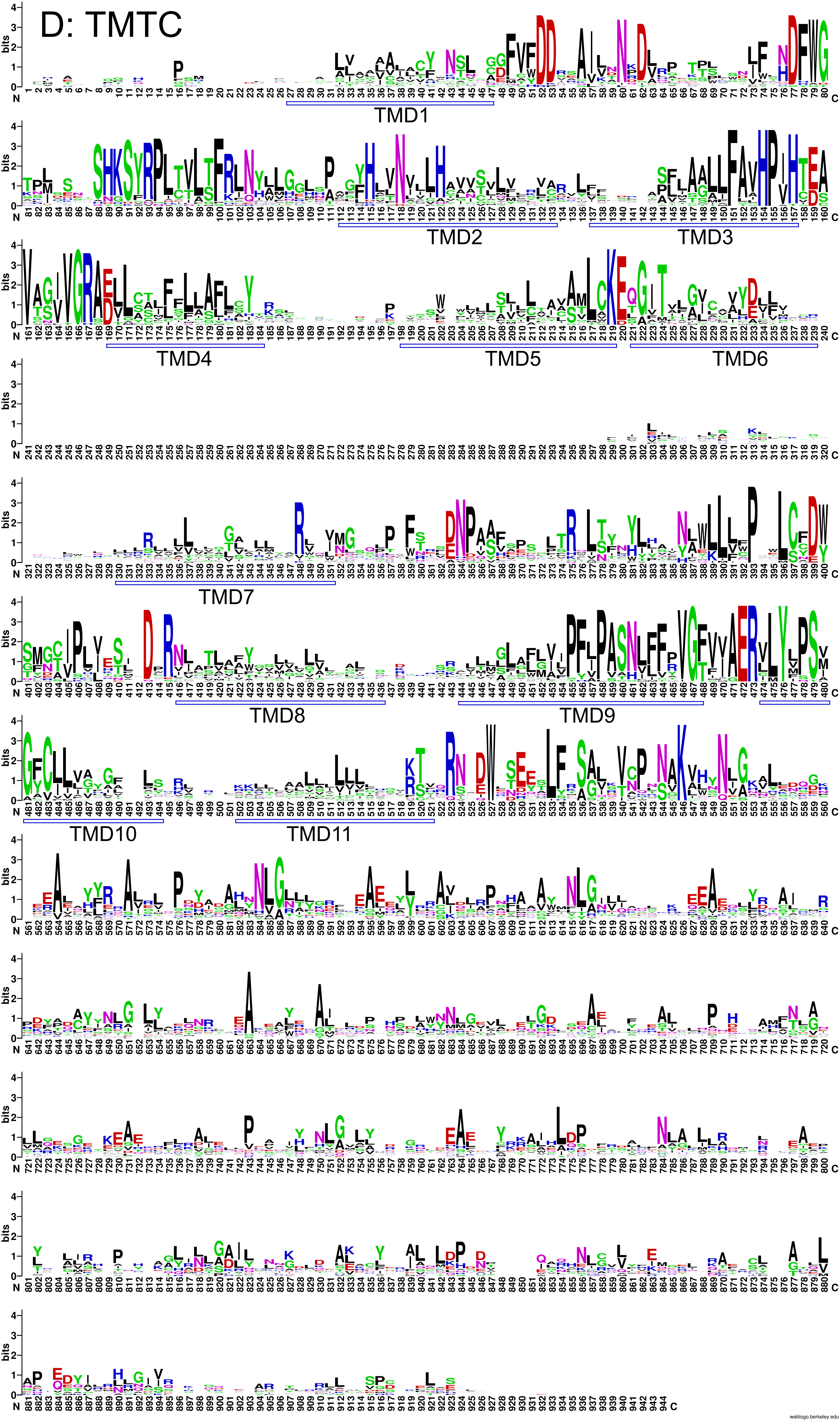
D: TMTC
bits
TMD1
TMD2
TMD3
TMD4
TMD5
TMD6
TMD7
TMD8
TMD9
TMD10
TMD11
weblogo.berkeley.edu

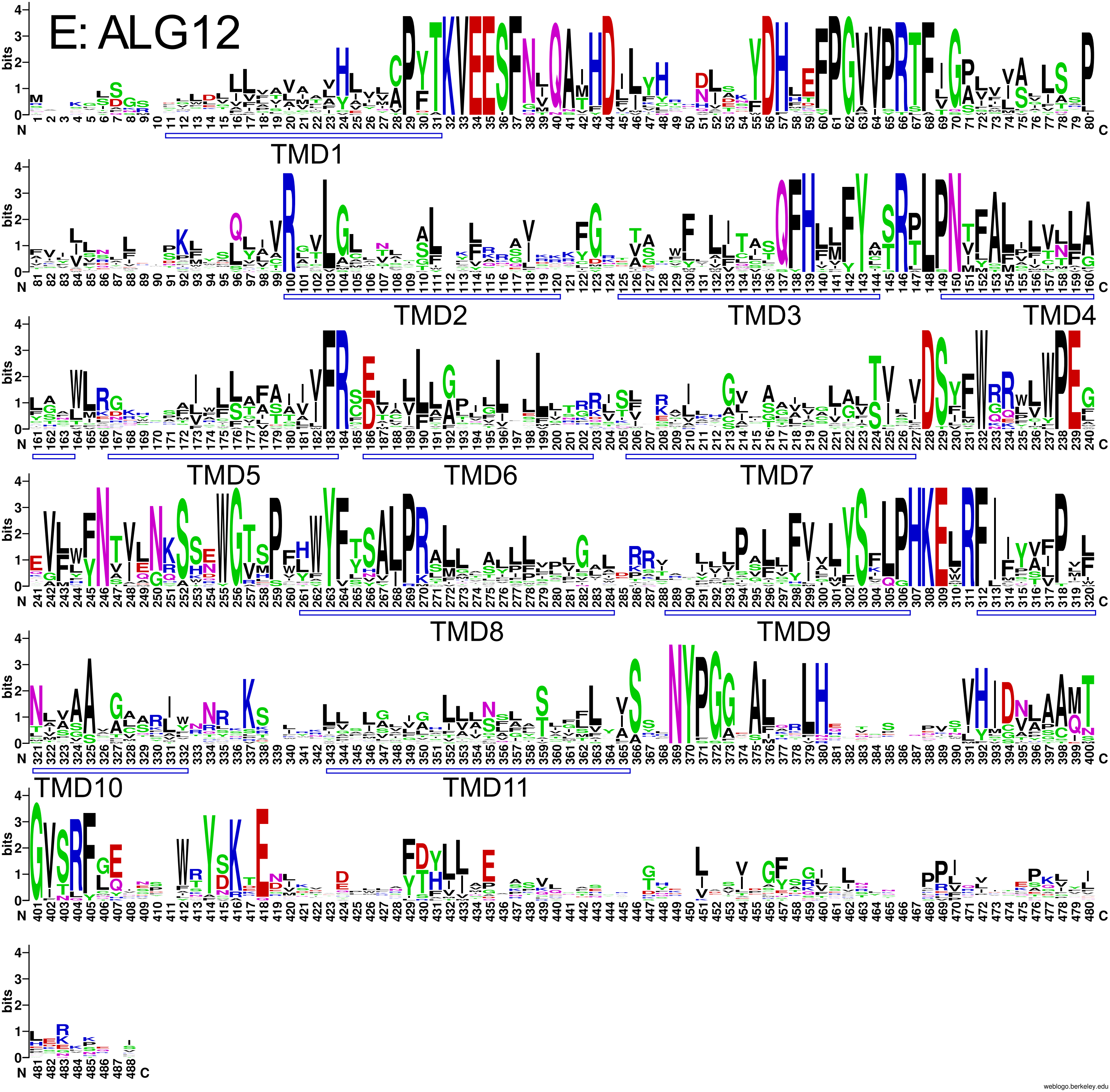

E: ALG12
bits
TMD1
TMD2
TMD3
TMD4
TMD5
TMD6
TMD7
TMD8
TMD9
TMD10
TMD11
weblogo.berkeley.edu

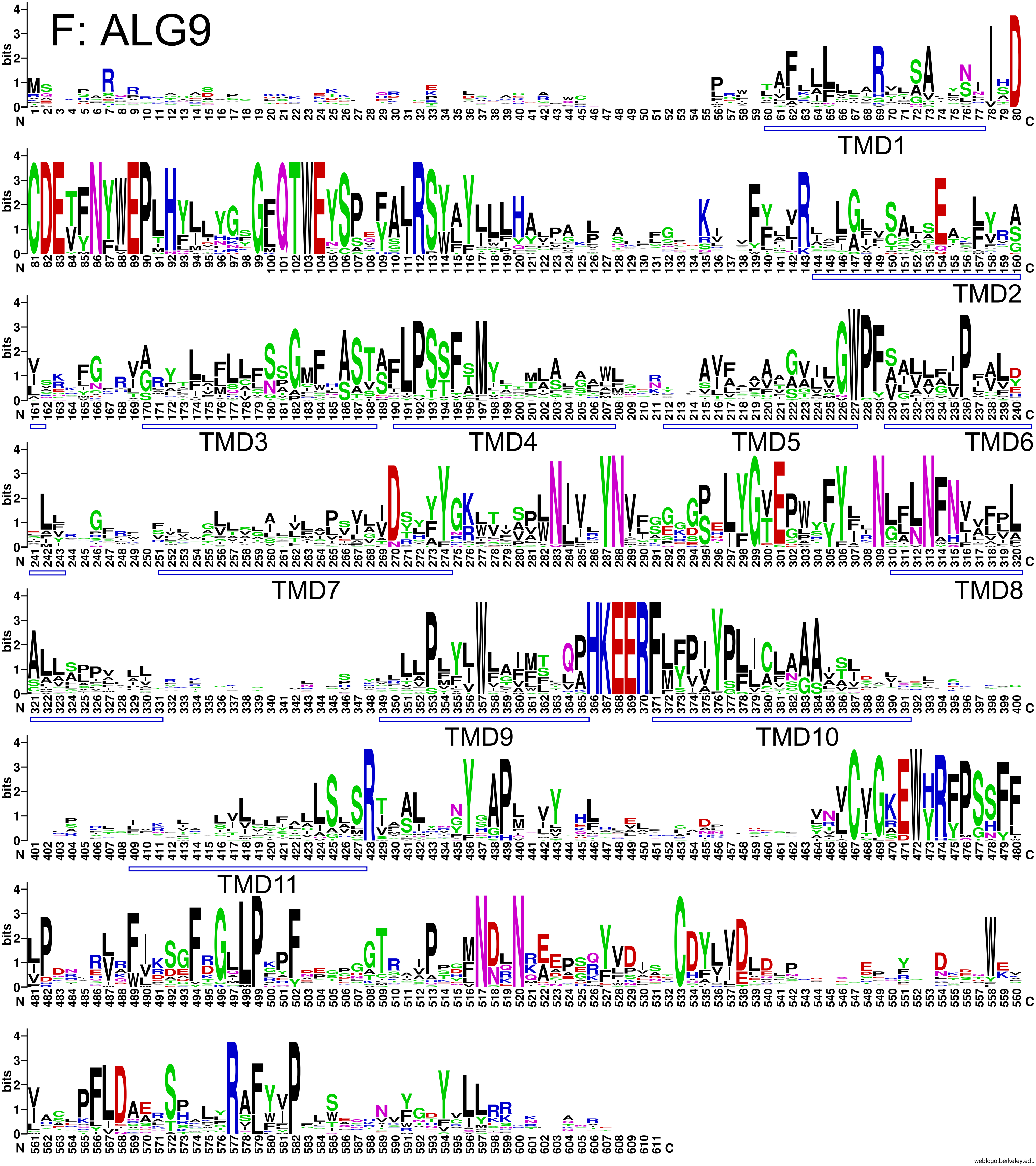
F: ALG9
bits
TMD1
TMD2
TMD3
TMD4
TMD5
TMD6
TMD7
TMD8
TMD9
TMD10
TMD11
weblogo.berkeley.edu

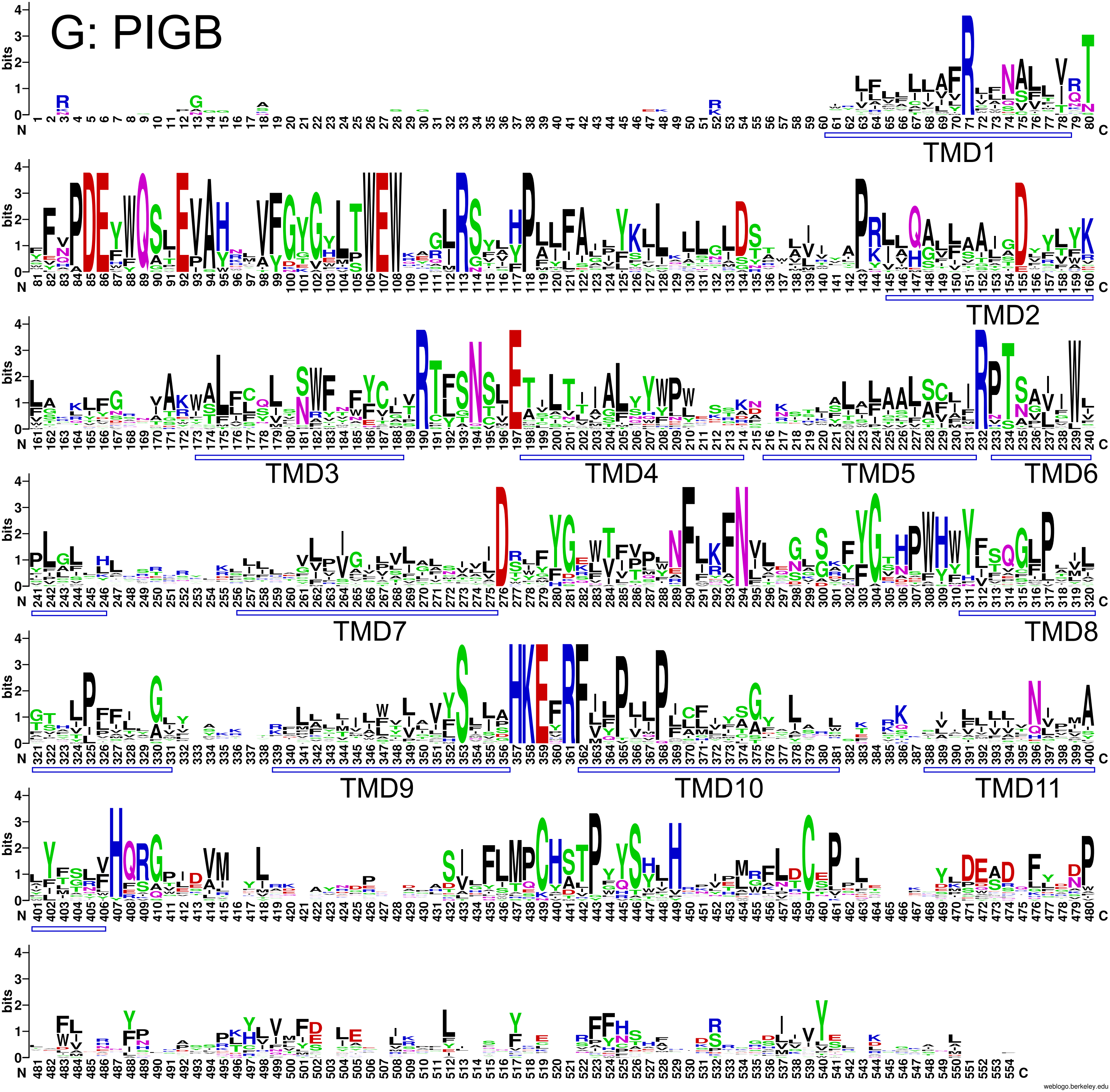
G: PIGB
bits
TMD1
TMD2
TMD3
TMD4
TMD5
TMD6
TMD7
TMD8
TMD9
TMD10
TMD11
weblogo.berkeley.edu

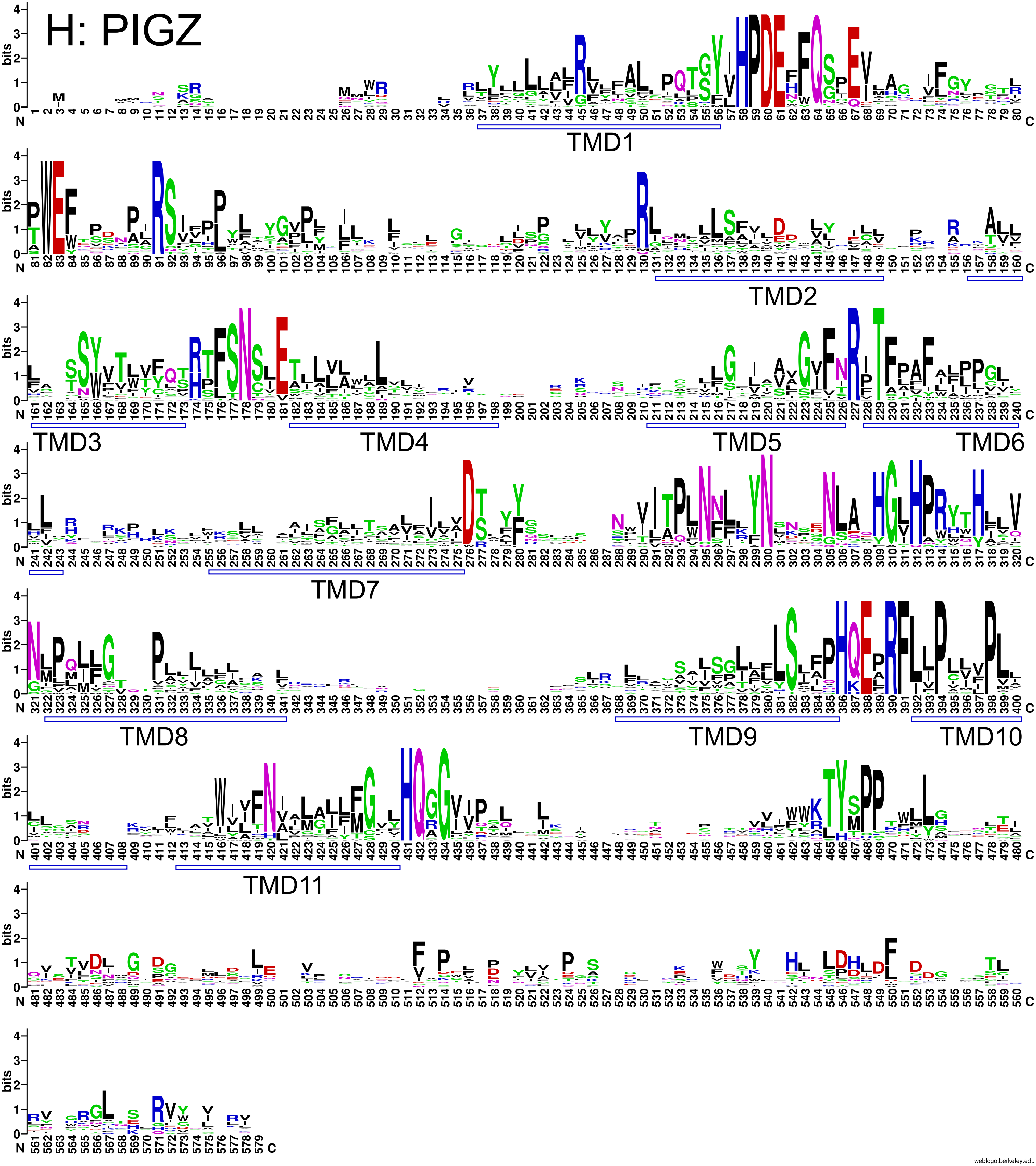
H: PIGZ
bits
TMD1
TMD2
TMD3
TMD4
TMD5
TMD6
TMD7
TMD8
TMD9
TMD10
TMD11
weblogo.berkeley.edu

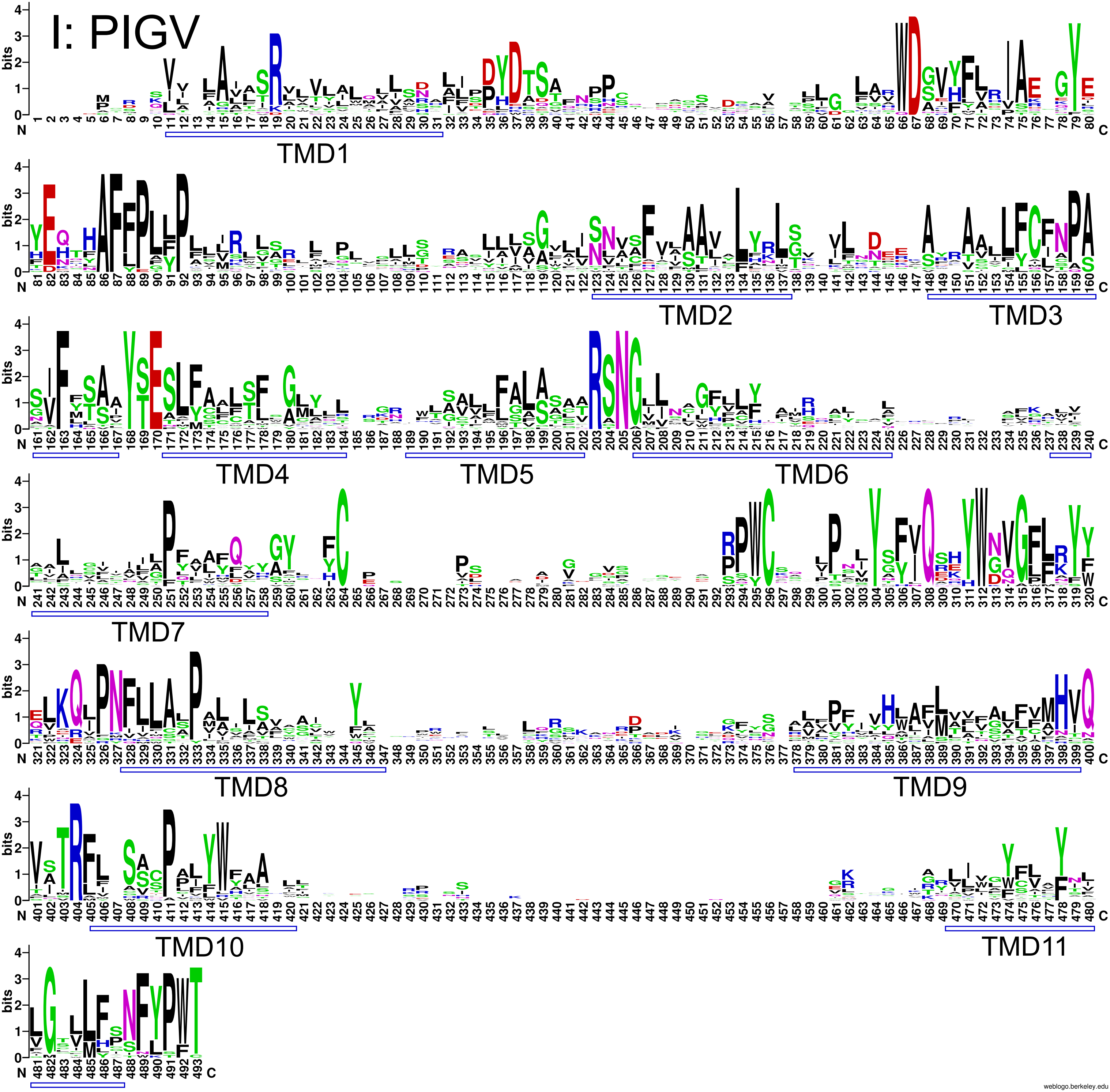
I: PIGV
bits
N
C
TMD1
TMD2
TMD3
TMD4
TMD5
TMD6
TMD7
TMD8
TMD9
TMD10
TMD11
weblogo.berkeley.edu

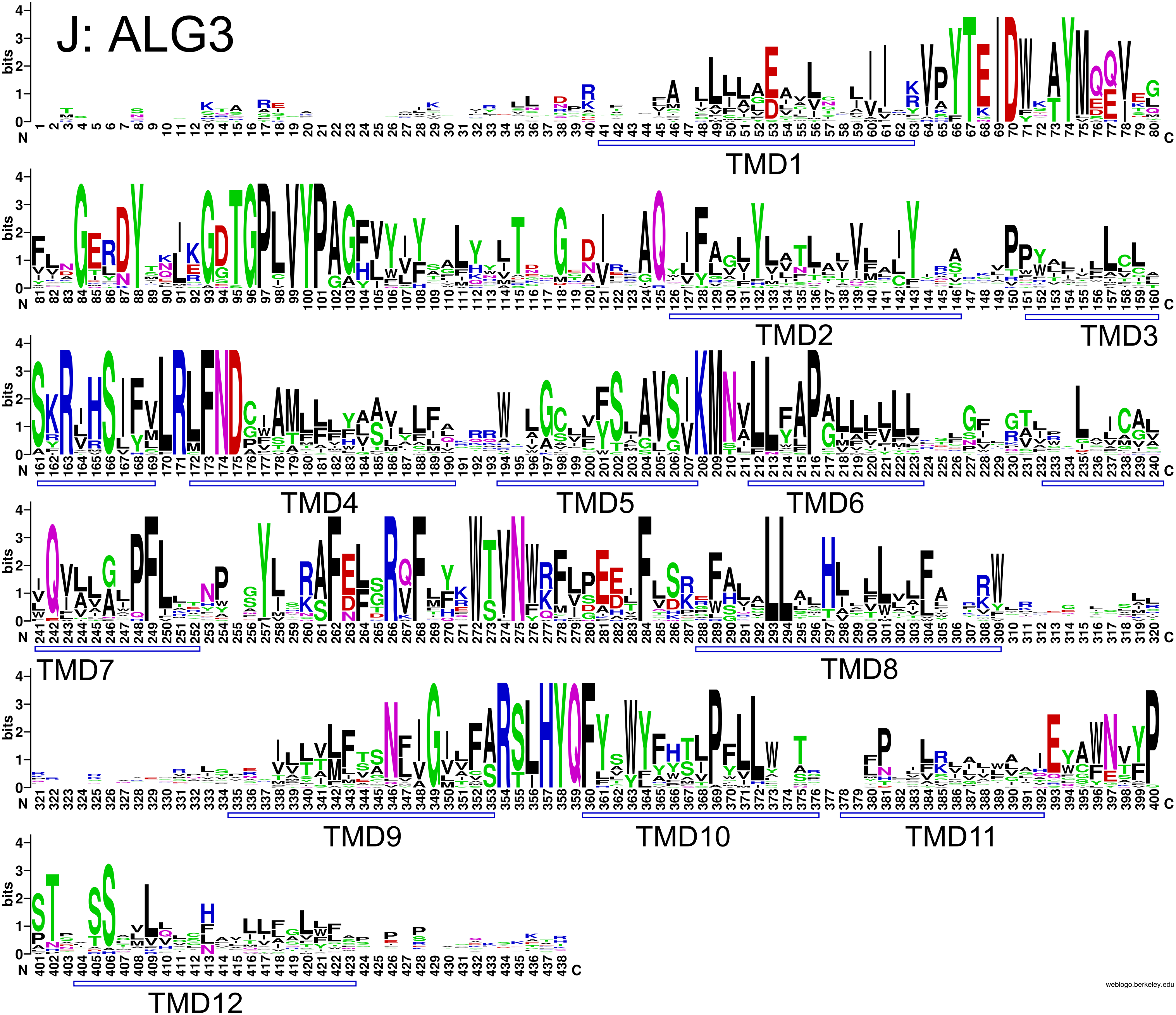
J: ALG3
bits
TMD1
TMD2
TMD3
TMD4
TMD5
TMD6
TMD7
TMD8
TMD9
TMD10
TMD11
TMD12
weblogo.berkeley.edu

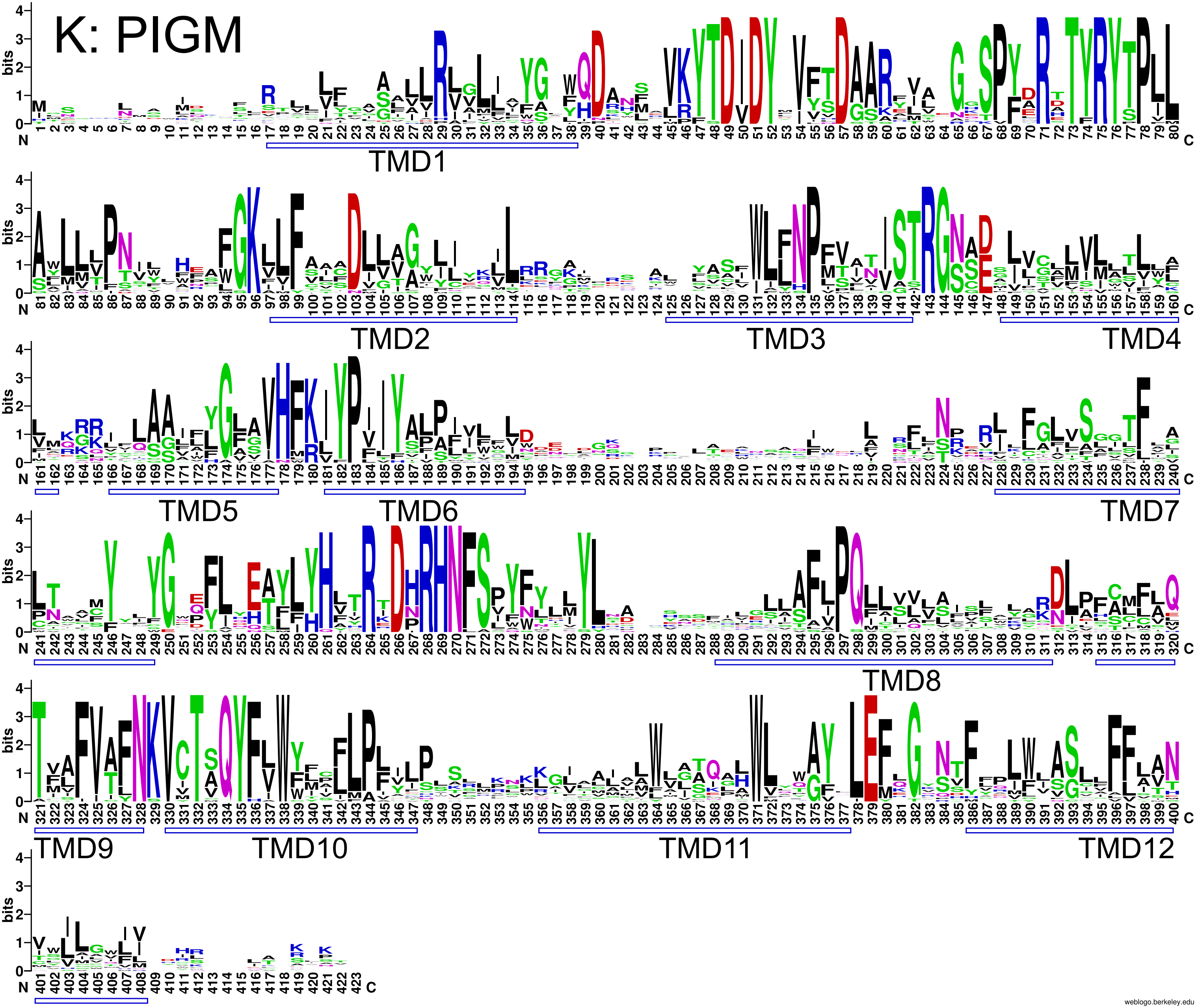
K: PIGM
bits
TMD1
TMD2
TMD3
TMD4
TMD5
TMD6
TMD7
TMD8
TMD9
TMD10
TMD11
TMD12
weblogo.berkeley.edu

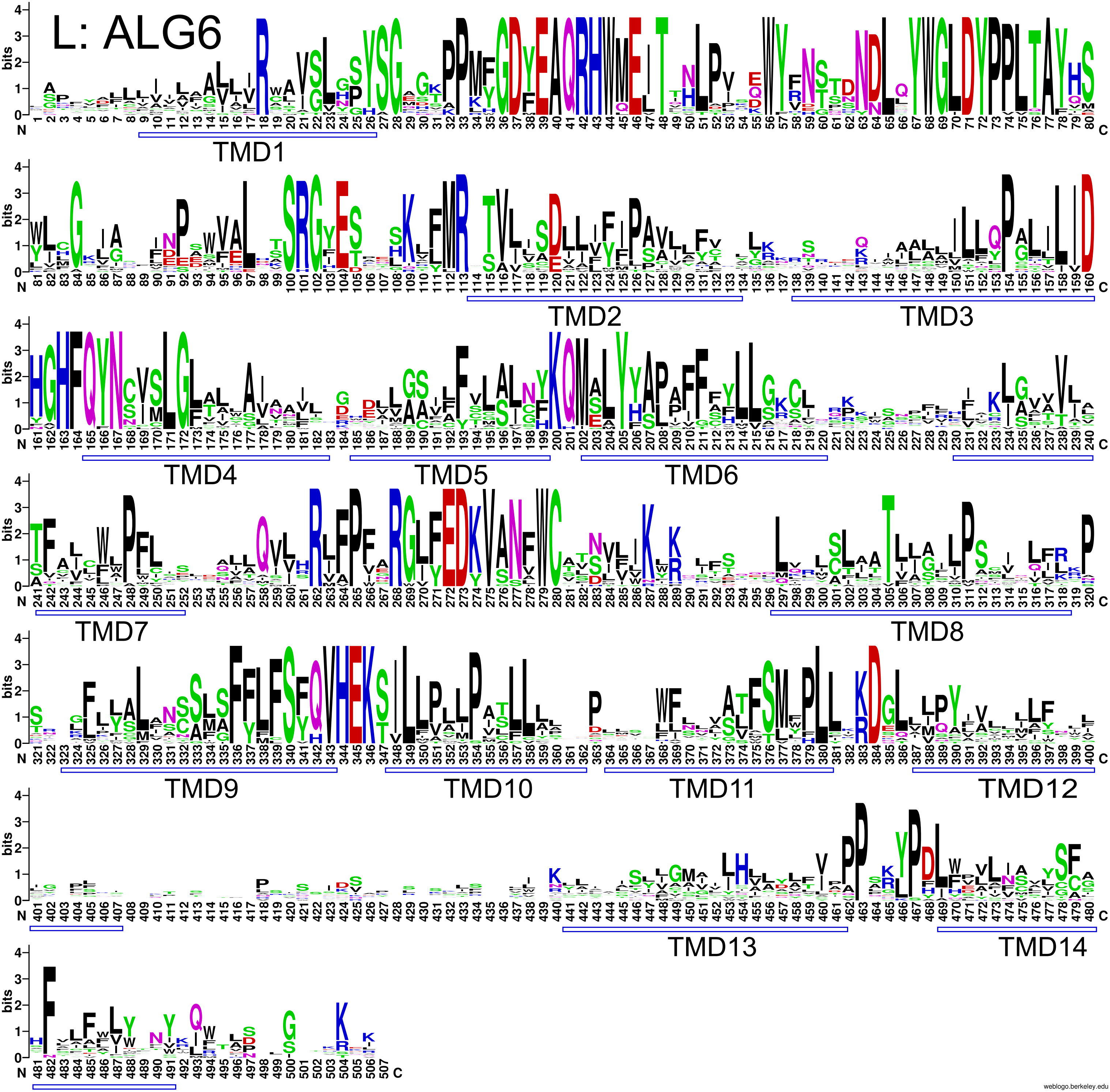
L: ALG6
bits
N
C
TMD1
TMD2
TMD3
TMD4
TMD5
TMD6
TMD7
TMD8
TMD9
TMD10
TMD11
TMD12
TMD13
TMD14
weblogo.berkeley.edu

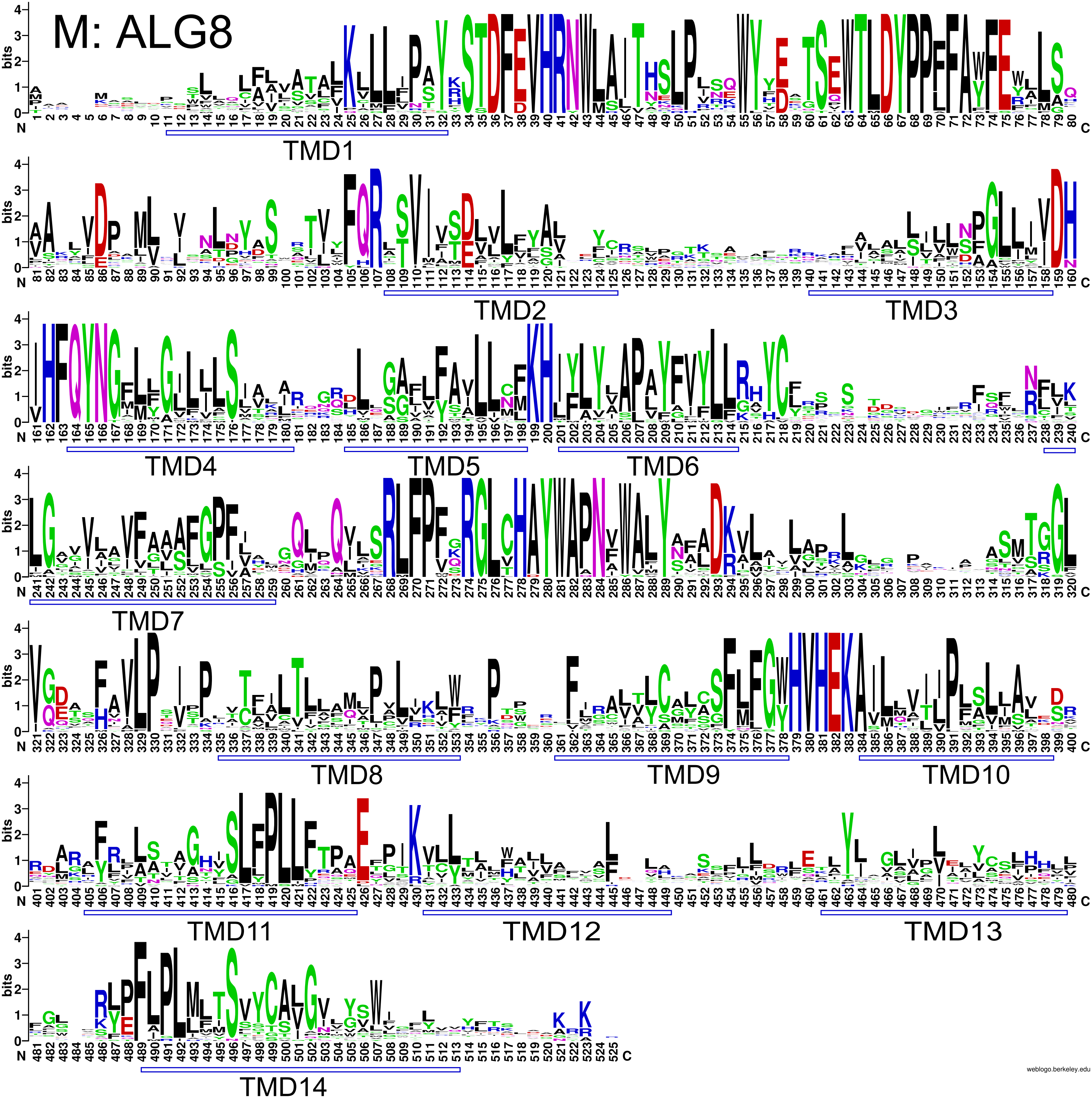
M: ALG8
bits
N
C
TMD1
TMD2
TMD3
TMD4
TMD5
TMD6
TMD7
TMD8
TMD9
TMD10
TMD11
TMD12
TMD13
TMD14
weblogo.berkeley.edu

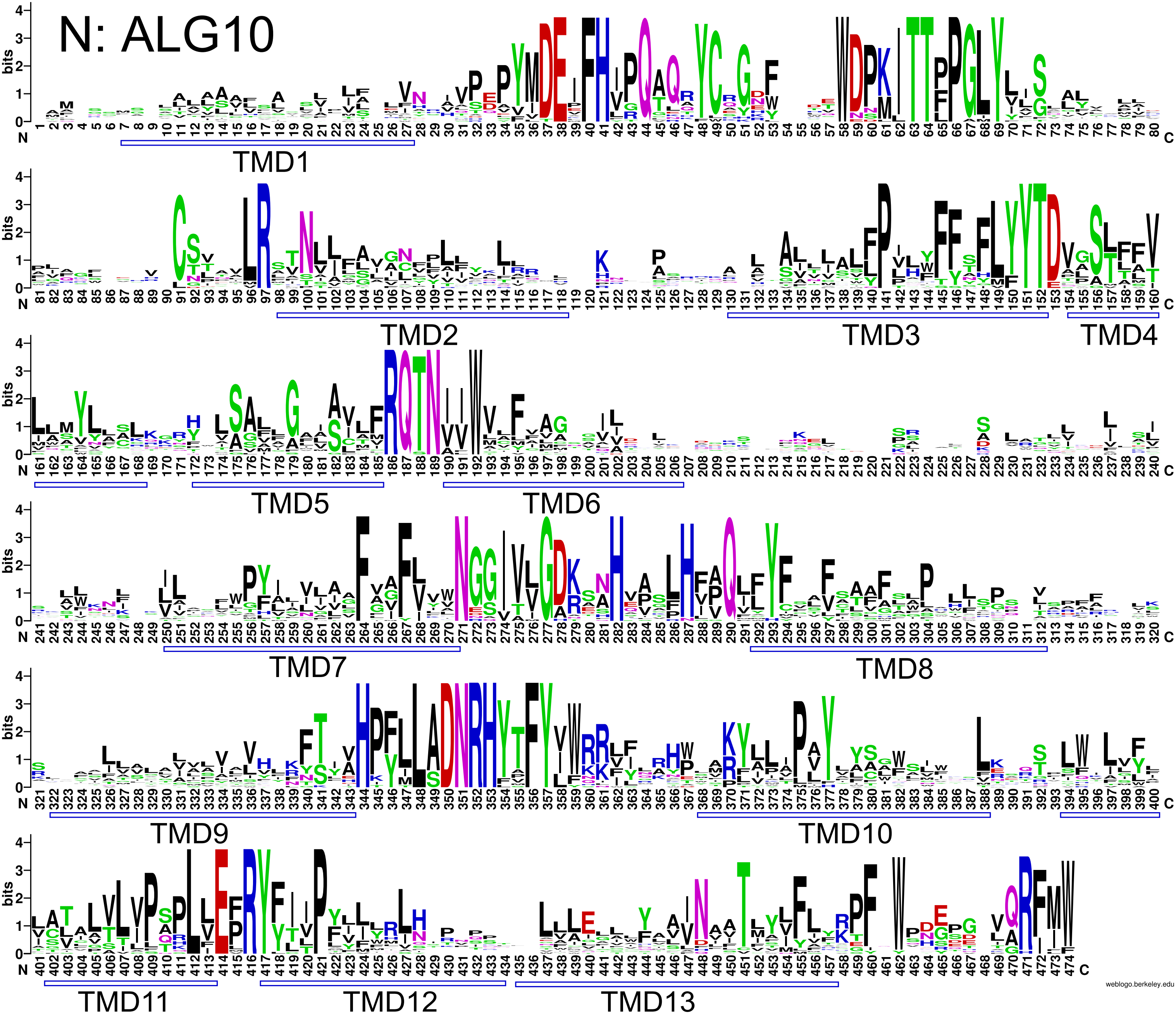

N: ALG10
bits
TMD1
TMD2
TMD3
TMD4
TMD5
TMD6
TMD7
TMD8
TMD9
TMD10
TMD11
TMD12
TMD13
weblogo.berkeley.edu

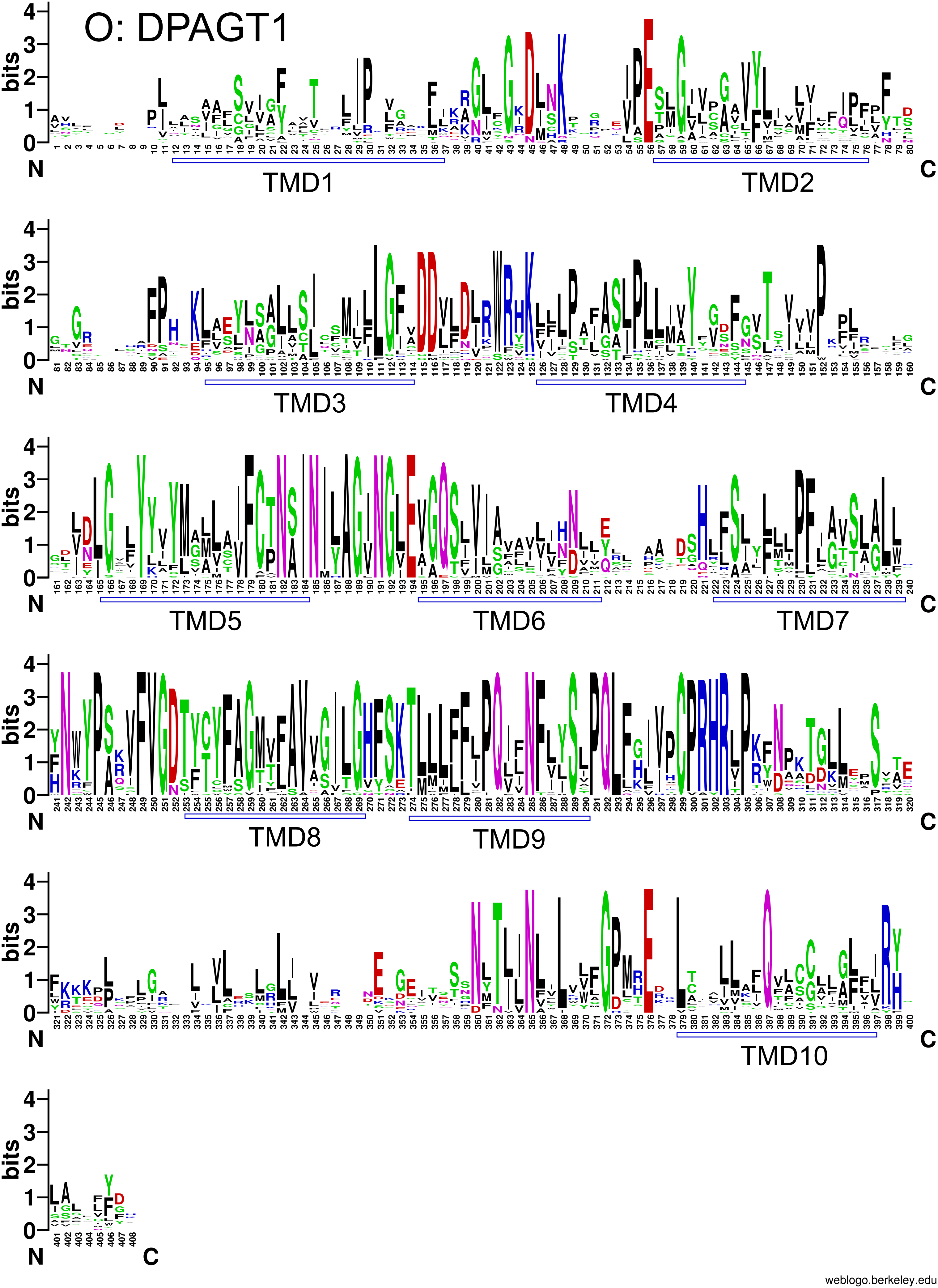
O: DPAGT1
bits
N
C
TMD1
TMD2
TMD3
TMD4
TMD5
TMD6
TMD7
TMD8
TMD9
TMD10
weblogo.berkeley.edu
